# Reversible writing/deleting of magnetic skyrmions through hydrogen adsorption/desorption

**DOI:** 10.1038/s41467-022-28968-4

**Published:** 2022-03-15

**Authors:** Gong Chen, Colin Ophus, Alberto Quintana, Heeyoung Kwon, Changyeon Won, Haifeng Ding, Yizheng Wu, Andreas K. Schmid, Kai Liu

**Affiliations:** 1 Physics Department, Georgetown University, Washington, DC 20057 USA; 2 Physics Department, University of California, Davis, CA 95616 USA; 3 NCEM, Molecular Foundry, Lawrence Berkeley National Laboratory, Berkeley, CA 94720 USA; 4 Center for Spintronics, Korea Institute of Science and Technology, Seoul, 02792 South Korea; 5 Department of Physics, Kyung Hee University, Seoul, 02447 South Korea; 6 National Laboratory of Solid State Microstructures, Department of Physics and Collaborative Innovation Center of Advanced Microstructures, Nanjing University, 22 Hankou Road, Nanjing, 210093 PR China; 7 Department of Physics, State Key Laboratory of Surface Physics and Advanced Materials Laboratory, Fudan University, Shanghai, 200433 PR China

**Keywords:** Surfaces, interfaces and thin films, Spintronics

## Abstract

Magnetic skyrmions are topologically nontrivial spin textures with envisioned applications in energy-efficient magnetic information storage. Toggling the presence of magnetic skyrmions via writing/deleting processes is essential for spintronics applications, which usually require the application of a magnetic field, a gate voltage or an electric current. Here we demonstrate the reversible field-free writing/deleting of skyrmions at room temperature, via hydrogen chemisorption/desorption on the surface of Ni and Co films. Supported by Monte-Carlo simulations, the skyrmion creation/annihilation is attributed to the hydrogen-induced magnetic anisotropy change on ferromagnetic surfaces. We also demonstrate the role of hydrogen and oxygen on magnetic anisotropy and skyrmion deletion on other magnetic surfaces. Our results open up new possibilities for designing skyrmionic and magneto-ionic devices.

## Abstract (web summary)

To use skyrmions to store information, an effective method for writing and deleting them is required. Here, Chen et al demonstrate the writing and deleting of skyrmions at room temperature by using hydrogen adsorption to change the magnetic anisotropy of the metallic multilayer hosting the skyrmions.

## Introduction

Magnetic skyrmions are bubble-like topological spin textures, characterized by a topological charge (or skyrmion number)^1,2^. One of the main mechanisms to stabilize skyrmions is the Dzyaloshinskii–Moriya interaction (DMI)^3,4^. The DMI only occurs under conditions where inversion symmetry is broken, for example in bulk B20 compounds^5^ or in thin films^6^, and skyrmions have been observed experimentally in many of these systems^2,7^. Due to their topologically protected spin configurations magnetic skyrmions have potential to be used as information carriers in spintronic applications, such as skyrmion-based memory^8,9^, logic devices^10^ or artificial neurons^11^. They may also find applications in more complex device architectures such as 3-dimensional (3D) racetrack memories^12^ or interconnected networks^13^. Being able to create and annihilate skyrmions conveniently is a key step towards achieving skyrmion-based spintronic devices. So far, writing/deleting of skyrmions has been done primarily using an applied magnetic field, spin-polarized current injection^14–18^, or applied gate voltage^19–22^. Recently, laser light pulses^23^ and thermal excitation^24^ have also been shown to generate skyrmions. In most of these approaches, the writing/deleting of skyrmions is realized by overcoming a finite energy barrier with the stimuli mentioned above^7,14–22^, where two local minima separated by the energy barrier correspond to the presence/absence of skyrmions, respectively. Exploring new approaches to write/delete skyrmions, particularly in a contactless manner, is both fundamentally interesting and practically important for device applications.

Manipulating magnetic materials and structures with light elements such as hydrogen or oxygen is an effective way to tailor their properties in field-free conditions. For instance, hydrogen absorption has been used to alter magnetic properties in thin films, including magnetic moment^25^, exchange coupling^26^, and anisotropy^27^. It was also shown to prompt the formation of a magnetic skyrmion phase in the Fe/Ir(111) system in external magnetic fields at 4.2 K^28^. In these prior observations, the altered magnetic properties were attributed to *absorption* of hydrogen into the bulk of the materials. On the other hand, *chemisorption* of hydrogen or oxygen on metal surfaces, limited to surface adsorption without penetrating into the metal interior^29^, has been shown to induce DMI^30,31^ and allow the tuning of magnetic anisotropy^32^. Understanding adsorbate induced magnetic properties is particularly relevant to the emerging field of magneto-ionics, where oxygen, hydrogen, or nitrogen ions can be driven to/from interfaces via gate voltage, enabling the reversible tuning of magnetic properties such as magnetic anisotropy and magnetization^33–37^. Combining the exciting promise of skyrmion-based spintronics and the field of light element-based magneto-ionics motivates the search for ways to control skyrmion properties through chemisorption.

In this paper, we report reversible hydrogen-driven writing/deleting of skyrmions in Ni/Co/Pd/W(110) multilayers at room temperature. Using spin-polarized low-energy electron microscopy (SPLEEM), skyrmion creation and annihilation is observed during hydrogen chemisorption/desorption cycles. We show that the adsorption of hydrogen on the surface of Ni/Co/Pd/W(110) multilayers changes the balance of magnetic energy contributions, particularly the magnetic anisotropy, which in turn drives the skyrmion creation/annihilation as the energy landscape evolves. Using SPLEEM for magnetization vector mapping we resolve the spin structure of the written skyrmions and show that they are left-handed hedgehog Néel-type. Monte-Carlo simulations support our interpretation attributing the reversible skyrmion writing and deleting to anisotropy changes. The roles of hydrogen and oxygen on magnetic anisotropy and skyrmion deletion on other magnetic surfaces are also demonstrated. Such ambient temperature reversible skyrmion operations in the absence of magnetic field, gate voltage or electric current provide new paths for the design of skyrmion-based spintronics and magneto-ionic devices.

## Results

### Hydrogen-induced reversible change of magnetic anisotropy and domain structure

**Fig. 1:**
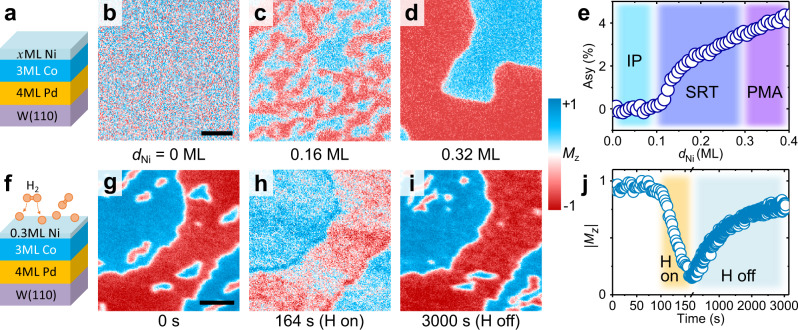
Hydrogen-induced magnetic anisotropy in Ni/Co/Pd/W system. **a** Sketch of the sample structure. **b**–**d** Ni thickness *d*_Ni_ dependent SPLEEM images with out-of-plane sensitivity in Ni/3 ML Co/4 ML Pd/W(110) system, showing evolution of perpendicularly magnetized domains. Scale bar 1 μm. **e** *d*_Ni_ dependent electron reflectivity spin-asymmetry of perpendicularly magnetized domains. IP, SRT, PMA stand for in-plane, spin reorientation transition and perpendicular magnetic anisotropy, respectively. **f**–**i** Hydrogen-induced evolution of domains with out-of-plane sensitivity. Scale bar 1 μm, indicating in-plane anisotropy induced by $\sim$0.7 Langmuir hydrogen on 0.3 ML Ni/3 ML Co/4 ML Pd/W(110) (panels **g** and **h**), and recovered perpendicularly magnetized domains upon hydrogen desorption (panel **i**). **j** Time-dependent switching of out-of-plane magnetization ($\left|{M}_{{{{{{\rm{z}}}}}}}\right|$) in one hydrogen ON/OFF cycle.

One effective way to tailor magnetic domains is the control of magnetic anisotropy, especially near a spin reorientation transition (SRT), where domain patterns are very sensitive to small changes of magnetic anisotropy^38^. In our system of Ni/Co/Pd/W(110) (see Methods), the effective magnetic anisotropy can be tuned by adjusting the Ni layer thicknesses ${d}_{{{{{{\rm{Ni}}}}}}}$ and, as long as the Co layer thickness is in the range of a few monolayer (ML), two typical SRTs occur, similar to other systems with two SRTs^39^. The first SRT from in-plane to out-of-plane appears with the deposition of a fraction of 1 ML of Ni, and the second SRT from out-of-plane to in-plane happens at a Ni thickness of about 2–3 ML. Using SPLEEM, we start by observing the evolution of magnetic domain patterns as a function of Ni film thickness, where ${d}_{{{{{{\rm{Ni}}}}}}}$ < 1 ML (Fig. 1a–d), which allows us to prepare samples near the first SRT. When Ni deposition is stopped right after the SRT, perpendicularly magnetized domains are observed in the test sample (Fig. 1d, e).

Chemisorbed hydrogen is subsequently added to the metal surface by dissociative adsorption of high-purity hydrogen leaked into the ultra-high vacuum chamber (Methods). Experimental studies^29,40^ and density functional theory (DFT) calculations^41,42^ have previously shown that the hydrogen atoms adsorb on the top surface and that diffusion into subsurface binding sites is kinetically hindered by the presence of a chemisorption energy well on both Ni and Co surfaces. On the fcc(111) - like Ni/Co surface, the hydrogen atoms are expected to occupy three-fold hollow sites, in the same binding geometry as on the close-packed pure Ni^43^ and Co surfaces^42^. The evolution of magnetic domains is monitored in real-time with the microscope aligned for out-of-plane magnetization sensitivity. Upon exposure of $\sim$0.7 Langmuir hydrogen on the sample with out-of-plane magnetized domains (Fig. 1f, g), the out-of-plane magnetization component of the domains becomes significantly smaller, i.e., ~10–20% of the initial $\left|{M}_{{{{{{\rm{z}}}}}}}\right|$, as shown in Fig. 1h, indicating that chemisorbed hydrogen induces an in-plane anisotropy. Note that the surface only contains a fraction of a monolayer (0.3 ML) of Ni, besides the chemisorption on the Ni, it is plausible to attribute the chemisorbed hydrogen induced in-plane anisotropy to the binding of hydrogen to the Co sites. This is consistent with the observation of hydrogen adsorption induced in-plane anisotropy on Co/Ru(0001)^32^, as well as with the in-plane anisotropy induced in the Pt/Co/GdO_*x*_ system via magneto-ionic proton transport^36^.

Once the hydrogen flux is turned off, the domains gradually return to perpendicular magnetization as hydrogen desorbs at room temperature (Fig. 1i). The time-dependent evolution of out-of-plane magnetic contrast $\left|{M}_{{{{{{\rm{z}}}}}}}\right|$ is plotted in Fig. 1j, showing the reversible hydrogen-induced anisotropy change during the chemisorption/desorption cycle. The reversible hydrogen chemisorption/desorption on Ni/Co surfaces is also supported by work function measurements^31^ (Supplementary Fig. S1). The entire cycle occurs at room temperature without heating or cooling, which suggests that the magnitude of the binding energy of hydrogen on Ni/Co surfaces is in an experimentally convenient range for enabling the reversible chemisorption/desorption of hydrogen. Other systems where reversible control of magnetic anisotropy was realized by adsorption/desorption of hydrogen including Ni/Cu(001)^27^ and Co/Ru(0001)^32^ required heating for hydrogen desorption, which may trigger temperature-induced changes of the micromagnetic structure.

### Resolving hydrogen-induced hedgehog skyrmion

**Fig. 2:**
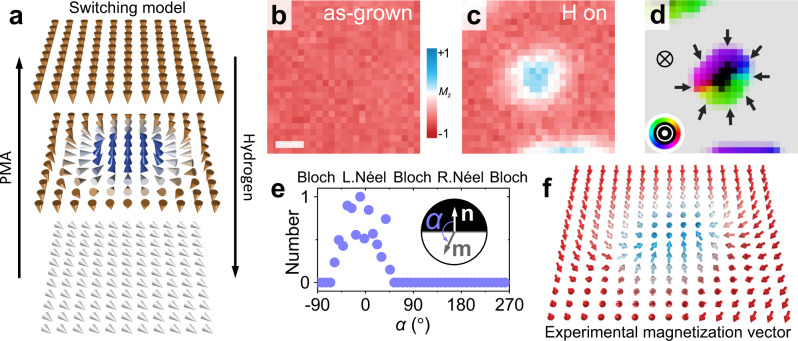
Hydrogen-induced skyrmion writing. **a** Perspective sketches of magnetization near spin reorientation transition in Ni/Co/Pd/W(110). **b** SPLEEM image of Ni/Co/Pd/W(110) (near SRT) on a local −*M*_z_ domain. Scale bar is 100 nm. **c** SPLEEM image at the same place as in panel **b** after 0.9 Langmuir hydrogen exposure, showing a bubble-like domain with +*M*_z_. **d** Compound SPLEEM image resolving the bubble-like domain as a skyrmion. Black/light gray represent up/down magnetization of the perpendicularly magnetized domains (see symbols), respectively, and color wheel shows in-plane magnetization direction within domain walls (also shown by black arrows). **e** Histogram of angle $\alpha$ between magnetization **m** and boundary normal **n**, measured pixel-by-pixel along domain boundaries, indicating left-handed chiral Néel walls. **f** Experimentally determined arrows-array representation of the skyrmion structure over a $200\times 200{{{{{{\rm{nm}}}}}}}^{2}$ area, each arrow shows the magnetization vector direction of one pixel in panel **d**.

The fact that chemisorbed hydrogen induces in-plane anisotropy on the Ni/Co surface allows us to tune the effective anisotropy near the SRT and therefore tailor the domain configurations in a controllable way (Fig. 2a). Magnetic films with perpendicular magnetic anisotropy (PMA) commonly form labyrinth (or stripe) domain structures near SRTs, which respond to changes of magnetic anisotropy by varying the domain width, i.e., the domain boundary density. Equilibrium phases composed of arrays of magnetic skyrmions (bubbles) are also possible, particularly under conditions where asymmetric +*M*_z_ and −*M*_z_ magnetized area fractions are stabilized either via external magnetic field, or via effective magnetic field from interlayer exchange coupling^44^ or exchange bias^22^, or in some cases without those driving forces (Supplementary Fig. S2). In the Ni/Co/Pd/W(110) system, we have observed the evolution from in-plane magnetization to skyrmions (bubbles) during the film growth in the absence of magnetic field (Supplementary Fig. S3). To demonstrate the hydrogen-induced skyrmion creation, a domain that is out-of-plane magnetized with relatively weak PMA, i.e., close to SRT, is prepared by monitoring the magnetization using SPLEEM during Ni growth (Fig. 2b). Within this uniformly magnetized domain, during the exposure to $\sim$0.9 Langmuir hydrogen, a bubble-like domain appears (Fig. 2c). We note that the chemisorption of hydrogen also induces finite DMI^31^, however the DMI change induced by 0.9 Langmuir in the Ni/Co/Pd/W system was quantified as ($0.01\pm 0.005$) meV/atom, which is roughly two orders of magnitude smaller than the effective DMI in the Ni/Co/Pd/W multilayer used here, with much larger Pd thickness^31^. Moreover, the effective DMI in this system is slightly weakened upon the chemisorption of hydrogen, i.e., hydrogen-induced right-handedness versus Pd-induced left-handedness, so the slight DMI change associated with hydrogen does not favor the formation of skyrmions (supported by the Monte-Carlo simulation, see Supplementary Fig. S4c, d). Therefore, the creation of the skyrmion can be attributed to the change of anisotropy.

Using SPLEEM we can image the three Cartesian components of the magnetization vector, thus mapping the spin-vector structures of domains in thin films^45^. Such a magnetization vector map is shown in the compound SPLEEM image (Fig. 2d). The black skyrmion core (+*M*_z_) within the gray out-of-plane magnetized domain (−*M*_z_) is surrounded by an in-plane magnetized skyrmion boundary where the local magnetization always points towards the core (see black arrows in Fig. 2d), showing that the observed bubble-like magnetic structure is a Néel-type skyrmion. For a statistically robust analysis of larger data sets, the angle $\alpha$ between the domain boundary normal and the local magnetization vector is measured at all image pixels along the domain boundary^46^. Plotting histograms of this angle $\alpha$ then permits unambiguous identification of the domain boundary chirality, as shown in Fig. 2e, where a single peak at $\alpha =0^\circ$ confirms left-handed magnetic chirality. This chirality is determined by the effective DMI in the Pd/W(110) system^30^ where, as a result of opposite signs of the DMI at the Co/W (right-handed) and Co/Pd (left-handed) interfaces, the sign and the magnitude of the DMI can be tuned by adjusting the Pd film thickness ${d}_{{{{{{\rm{Pd}}}}}}}$. The thickness ${d}_{{{{{{\rm{Pd}}}}}}}$ in this sample is much larger than the zero-DMI thickness where the left-handed DMI of Pd just compensates the right-handed DMI of the W(110) substrate, therefore the effective DMI in this sample is Pd-like, i.e., left-handed. To display the measured spin structure more clearly, the same image is plotted again in Fig. 2f as an arrows array, where the orientations of the arrows represent the magnetization directions measured at image pixels in the central $200\times 200{{{{{{\rm{nm}}}}}}}^{2}$ region of panel 2d, highlighting the experimentally measured spin structure of this hedgehog skyrmion.

### Reversible writing and deleting of skyrmions using hydrogen

**Fig. 3:**
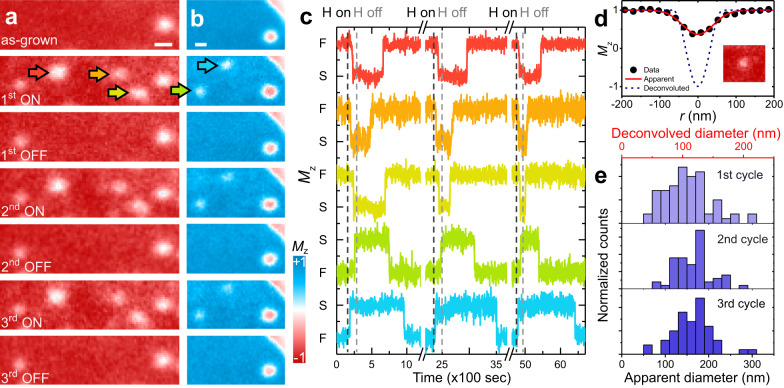
Reversible writing/deleting of magnetic skyrmions. **a** SPLEEM images of perpendicularly magnetized domains in Ni/Co/Pd/W(110), showing reversible skyrmion writing/deleting over three hydrogen ON/OFF cycles (indicated by the red, orange and yellow arrows), scale bar is 200 nm. **b** Similar time sequence as panel **a**, here on a +*M*_z_ domain (blue), showing reversible writing/deleting of two skyrmions (green and blue arrows), scale bar is 200 nm. **c** Time-dependent *M*_z_ evolution at the center of skyrmions highlighted by the arrows in panels **a** and **b** over three hydrogen ON/OFF cycles, showing reversible skyrmion switching between ferromagnetic state (F on *y* axis) and skyrmion state (S on *y* axis). Color of the curve corresponds to the arrow color. **d** *M*_z_ profile of a skyrmion (see inset) and its deconvolved profile in ideal imaging condition. **e** Histograms of skyrmion diameters (lower scale image-apparent, upper scale deconvolved measurements) over three cycles of H ON/OFF.

Observation of the reversible control of magnetic anisotropy via hydrogen chemisorption/desorption, as summarized in Fig. 1, together with the hydrogen-induced writing of skyrmions, as shown in Fig. 2, suggests the opportunity of creating/annihilating skyrmions via cycles of hydrogen chemisorption/desorption. A sample of Ni/Co/Pd/W(110) with weak PMA and some initial skyrmions was prepared, and Fig. 3a shows the evolution of its magnetization within a −*M*_z_ domain over three hydrogen ON/OFF cycles, where skyrmions are created in each H-ON state and annihilated in each H-OFF state. The total dose for each H-ON cycle is $\sim$0.9 Langmuir (5 × 10^−9^ torr hydrogen for 3 min). The duration of the hydrogen OFF cycles was chosen to be sufficiently long to allow spontaneous room temperature desorption of the hydrogen and recovery of the magnetization to nearly its original state. The hydrogen desorption rate, being a function of the hydrogen binding energy to the Ni/Co/Pd/W surface, is somewhat dependent on the sub-monolayer Ni film thickness^31,47^. We find that the skyrmions are mostly created/deleted at the same location on the film surface, which is likely related to small variations of anisotropy across the film. We speculate that the exact value of the PMA has fluctuations associated with details of atomic-scale surface and interface properties such as defects and step density^48^, and that areas featuring slightly reduced PMA may result in a higher probability for skyrmion creation; this hypothesis was tested in Monte-Carlo simulations described below. There are also some skyrmions already present prior to the introduction of hydrogen, which are not sensitive to hydrogen. We also show equivalent hydrogen ON/OFF cycles on a domain magnetized in the opposite direction, +*M*_z_ (Fig. 3b), where similar skyrmion switching is observed. Successful skyrmion switching on both +*M*_z_ and −*M*_z_ domains excludes any unidirectional driving force, such as a magnetic field.

The creation and annihilation times of these individual skyrmions were extracted from the microscopy data in Fig. 3a and b, and plotted in Fig. 3c. The measurable differences in the exact time at which individual skyrmions are turned on and off are likely related to the hypothesized landscape of PMA variations, and possibly to hydrogen adsorption/desorption rate variations associated with microscopic details of the Ni coverage. To measure the size of the skyrmions based on their magnetization profiles (Fig. 3d), we follow ref. ^49^ and start by assuming that observed skyrmion images represent convolutions of the skyrmions’ physical magnetization profiles and the resolution limit of the SPLEEM images. The instrumental blur is evident by observing how the regions of reversed magnetization in the skyrmion cores are clearly resolved in larger skyrmions and is progressively blurred as skyrmion size shrinks. Figure 3d shows an example of this deconvolution, where the red solid curve represents the apparent skyrmion profile as measured directly from the image and the blue dashed curve represents the estimate of its physical size based on deconvolving the image blur. Histograms of skyrmion sizes measured during three write/delete cycles are shown in Fig. 3e, where the lower scale indicates apparent diameters from the images and the upper scale, labeled ‘deconvolved diameter’, represents the results including deconvolution of image blur. These data indicate that the average diameter of these ensembles of skyrmions is of the order of $\sim$100 nm, with many skyrmions in the sub-100 nm size region. No significant correlation between the skyrmion diameter and the writing or deleting time is observed (Supplementary Fig. S5). Overall, the skyrmion lifetimes are roughly constant over three cycles (Supplementary Fig. S6), and observed slight lifetime variations seem to be stochastic, apparently as a result of the thermodynamics of the hydrogen chemisorption/desorption process. We note that theoretical modeling of similar PMA systems predicts that the response of domain patterns to anisotropy changes is expected to result in variation of the width of stripe domains^50^, while bubble domains are favored when the degeneracy of +*M*_z_ vs. −*M*_z_ magnetization is broken by a unidirectional driving force. This general picture suggests that a rich variety of additional applications of the hydrogen-induced skyrmion writing/deleting could be realized by adding applied magnetic fields or by introducing interlayer exchange coupling^44^ or exchange bias^22^.

### Monte-Carlo simulation

**Fig. 4:**
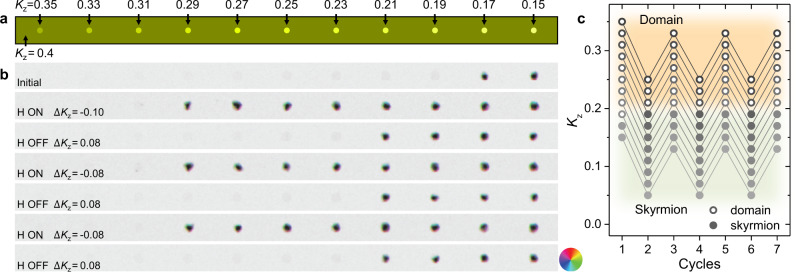
Monte-Carlo simulation of reversible writing/deleting of skyrmions via anisotropy change near SRT. **a** Sketch of the anisotropy landscape, highlighting pinning sites with various magnetic anisotropy (labeled on top). **b** Simulated domains over three anisotropy change cycles, showing reversible writing/deleting of skyrmions on the sites with *K*_z_ = 0.29, 0.27, 0.25 and 0.23. The amount of anisotropy change is indicated as $\triangle$*K*_z_. Exchange and DMI constants remain the same, where $J=1$, $\beta =0.3$ (see methods). Color wheel indicates the in-plane magnetization direction. **c** Anisotropy dependent single-domain (open circles) and skyrmion (gray circles) evolution, summarizing panel **b**.

Monte-Carlo simulations were performed to further understand the skyrmion writing/deleting process as a function of the parameters $J$, $\beta (=\big|{{{{{{\boldsymbol{\beta }}}}}}}_{{ij}}\big|)$, and ${K}_{{{{{{\rm{z}}}}}}}$, corresponding to exchange interaction, DMI, and magnetic anisotropy, respectively (see Methods). To simulate an out-of-plane magnetized film that is close to a SRT, an out-of-plane magnetized domain is initialized with small PMA *K*_z_ = +0.4, where the *K*_z_ value is normalized with respect to the exchange constant^51^. In addition, eleven pinning regions are simulated by locally reducing PMA in the range of 0.35 to 0.15 in steps of 0.02 (Fig. 4a). The role of the hydrogen chemisorption is simulated as a negative magnetic anisotropy shift of −0.10 on the entire area. The simulation shows that hydrogen chemisorption (i.e., anisotropy shift by −0.10) results in the creation of skyrmions at the pinning sites where *K*_z_ ranges from 0.29 to 0.19. At the regions with the lowest anisotropy (*K*_z_ = 0.17 and 0.15 in Fig. 4b) skyrmions remain stable with or without the simulated hydrogen adsorption. Because the chemisorbed hydrogen does not always fully desorb on the Ni/Co surface^31^, a partial recovery of magnetic anisotropy by +0.08 within the finite H-OFF time (Fig. 1j) was simulated, which showed that only those skyrmions with *K*_z_ = 0.29, 0.27, 0.25 and 0.23 were deleted. Additional cycles of anisotropy changes, varying the anisotropy by −0.08 (mimicking H-ON) and +0.08 (H-OFF), reproduce the reversible writing and deleting of skyrmions as was observed in the experiment (Fig. 3a). The simulations are summarized in Fig. 4c by plotting skyrmion presence (gray circles) and absence (open circles) in anisotropy versus H-cycle space, showing that the skyrmion switching occurs once the effective magnetic anisotropy crosses a boundary near *K*_z_ = +0.2 in the energy landscape between the two phases. Incidentally, the sign of H-induced DMI supresses the formation of skyrmions (Supplementary Fig. S4c, d), and additional simulations (Supplementary Fig. S4b) show that this small DMI variation is insufficient to affect the simulation results in Fig. 4b.

### Possible writing/deleting of skyrmions in other systems

**Fig. 5:**
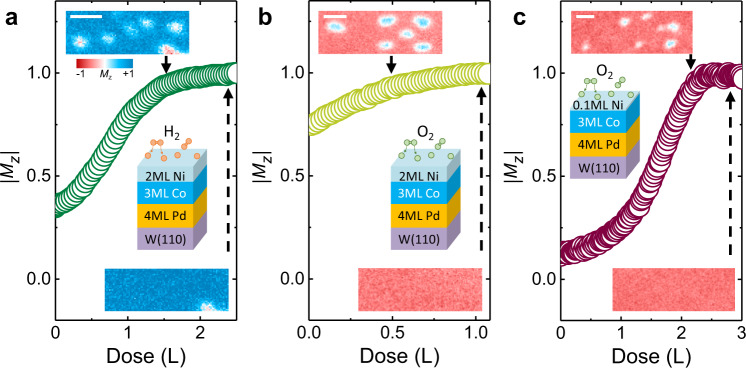
The role of chemisorbed hydrogen and oxygen on magnetic anisotropy and skyrmions on surfaces of other magnetic layers. **a** Hydrogen-induced anisotropy change (*M*_z_, integrated from SPLEEM images in insets) and skyrmion deleting (insets) on surface of 2 ML Ni/3 ML Co/4 ML Pd/W(110). The hydrogen pressure is 1.5 × 10^−8^ torr. The scale bar in the inset is 500 nm. **b**, **c** Oxygen induced anisotropy change (*M*_z_) and skyrmion deleting (insets) on surface of 2 ML Ni/3 ML Co/4 ML Pd/W(110) (panel **b**) and 0.1 ML Ni/3 ML Co/4 ML Pd/W(110) (panel **c**). Scale bars in the insets are 500 nm. The oxygen pressure is 2 × 10^−9^ torr in panel **b** and 1 × 10^−8^ torr in panel **c**.

The simulation results suggest that writing/deleting of skyrmions via chemisorption-induced anisotropy may be a general approach. Thus it is useful to explore skyrmion writing/deleting in other systems as well, particularly in light of potential hydrogen and oxygen based applications in magneto-ionics^33–36^. Chemisorption of hydrogen occurs on surfaces of other metals^29^, e.g., on the Ni(111). Figure 5a shows the evolution of spin structures including skyrmions during hydrogen chemisorption on the surface of 2 ML Ni/3 ML Co/Pd/W(110), where the chemisorption is comparable to the bulk-Ni(111) case because of the much thicker Ni film^31^, in contrast to that shown in Figs. 1–3. It is interesting to point out that while chemisorbed hydrogen induces in-plane magnetic anisotropy on the Co-rich surface (see also the same trend on the close-packed Co surface in Co/Ru(0001)^32^), chemisorbed hydrogen *enhances* the PMA on the Ni(111) surface. Prior ab initio studies revealed that the hydrogen-induced anisotropy is a local electronic effect and not a strain effect, i.e., it results from the hybridization of the hydrogen and the Co atoms closest to the adsorbed hydrogen^32^. Chemisorption of oxygen also occurs on Ni and Co surfaces^30^. Here we test the role of oxygen chemisorption on the magnetic domain evolution, on a Co-rich surface in a 0.3 ML Ni/3 ML Co/Pd/W(110) sample, and on a pure Ni surface in a 2 ML Ni/3 ML Co/Pd/W(110) sample, respectively. We find that chemisorbed oxygen *enhances* the PMA in both the Co-rich and the Ni surfaces. In these three cases, we demonstrate that magnetic skyrmions can be *deleted* upon chemisorption (Fig. 5 insets) as a result of the greater PMA of the system, which is opposite to the case of hydrogen on Co-rich surface (Figs. 1–3). Note that in these cases we have not observed reversibility of skyrmion formation, partly as a result of kinetically less favorable conditions due to higher binding energies between adsorbates and surfaces. However, reversible skyrmion switching may be facilitated via magneto-ionic approaches, e.g., using a gate voltage^36^ as discussed further below.

## Discussion

The writing/deleting of magnetic skyrmions via adsorption/desorption is associated with the domain evolution in the equilibrium state as the energy landscape changes. In contrast to prior approaches of writing/deleting skyrmions by overcoming an energy barrier via other external stimuli, this approach may tailor the dynamics of skyrmion writing/deleting by fine tuning the energy landscape. This appraoch also adds a new degree of freedom to chiral spintronics, where spin textures may be controlled in a tunable and contactless way, without the need for electrical leads. This may be particularly relevant for three-dimensional information storage schemes involving complex architectures and large numbers of skyrmions, such as racetrack memories^12^ or interconnected networks^13^. This effect can also be readily integrated into magneto-ionic devices consisting of ferromagnet/reservoir heterostructures, where the adsorption/desorption takes place at buried interfaces^36^. For example, oxygen, hydrogen or other species may be stored in a reservoir layer, and subsequently driven into contact with a ferromagnetic layer, where chemisorption/desorption may occur at the ferromagnet/reservoir interface. It would be particularly attractive to reversibly control the magnetic anisotropy and interfacial DMI via chemisorption, and in turn skyrmion writing/deleting, especially given the high mobility of hydrogen in solids. Note that the essential adsorption/desorption processes may occur at the relevant ferromagnet interface with the reservoir layer, without penetrating inside the ferromagnet, thus enabling reversibility. In this case high switching speeds reaching <1 ns^52^ may be possible, as the ionic species only need to traverse atomic distances to trigger chemisorption/desorption under proper device design. Such skyrmion-based devices with magneto-ionic functionality may be used for magnetic memory and logic devices, as well as artificial synapses.

In summary, we report the writing/deleting of skyrmion via hydrogen chemisorption/desorption on the surface of ferromagnets at room temperature, in the absence of magnetic field, gate voltage or electric current. Magnetization vector imaging by SPLEEM shows that the hydrogen chemisorption induced skyrmions are of the hedgehog Néel-type and the diameter of the skyrmions can be down to sub-100 nm. The driving force is attributed to hydrogen chemisorption induced magnetic anisotropy changes, which is supported by Monte-Carlo simulations. We also explore extension of the mechanism to chemisorbed hydrogen and oxygen on both Ni and Co-rich surfaces. These results open up alternative approaches for designing skyrmion-based devices.

## Methods

### Sample preparation

The experiments were performed at the National Center for Electron Microscopy of the Lawrence Berkeley National Laboratory. Samples were grown under ultra-high vacuum conditions in the SPLEEM chamber, with a base pressure better than 4.0 × 10^−11^ torr. The W(110) substrate was prepared by cycles of flashing to 1950 °C in 3.0 × 10^−8^ torr oxygen until the surface was free of carbon, followed by a final flashing at the same temperature to remove oxygen. Ni, Co and Pd layers were deposited by means of physical vapor deposition from electron beam evaporators, while the substrate is held at room temperature. The film thicknesses of the metal layers were calibrated via oscillations of the low-energy electron microscopy (LEEM) intensity associated with layer-by-layer growth^30^. Hydrogen exposures were introduced by leaking hydrogen of 99.999% purity at a pressure of at $5\times {10}^{-9}\,{{{{{\rm{torr}}}}}}$ into the SPLEEM chamber. The reading of the hydrogen pressure on the ionization gauge was corrected by a factor of 0.46.

### Magnetic imaging and analysis

The real-space magnetic images were taken using the SPLEEM at the National Center for Electron Microscopy of the Lawrence Berkeley National Laboratory. The magnetic contrast is the asymmetry *A* of the spin-dependent reflectivities *I* between spin-polarized beams with up (${I}_{\uparrow }$) and down (${I}_{\downarrow }$) polarization, $A=({I}_{\uparrow }-{I}_{\downarrow })/({I}_{\uparrow }+{I}_{\downarrow })$. This asymmetry *A* is proportional to ${{{{{\bf{P}}}}}}\cdot {{{{{\bf{M}}}}}}$, where **P** is the spin polarization vector of the illumination electron beam and **M** is the magnetization vector. Therefore the Cartesian components *M*_x_, *M*_y_ and *M*_z_ of the magnetization can be obtained by setting the spin-polarization alignment along the *x*, *y*, *z* directions, respectively^45^. The energies of the incident electron beam were set to a value in the range of 5–6 eV to optimize the magnetic contrast for the Ni/Co/Pd/W(110) system with various film thicknesses. All images were measured with the samples held at room temperature. All the experiments were done in the absence of magnetic field.

Image drift-correction and denoising are applied on time-dependent SPLEEM image sequences. For small skyrmions, the minimum value of *M*_z_ does not reach −1 due to the limited image resolution. In all cases, we have estimated the ideal skyrmion profile by deconvolving the measured profile with the estimated instrument point spread function^49^. We use full-width-at-half-maximum of the skyrmion profile as the skyrmion diameter for both image-apparent and deconvolved values. The compound SPLEEM images are converted by combining three sets of *M*_x_, *M*_y_ and *M*_z_ SPLEEM images, where the color wheel represents in-plane magnetization directions, and gray values indicate the perpendicular magnetization component +*M*_z_ (black), −*M*_z_ (gray), respectively.

The time series data were analyzed using custom Matlab codes with three pre-processing steps. Histograms of the measured intensity were used to fit scaling coefficients to the *M*_z_ channel, such that the two domains had mean values of −1 and +1, and then applied this scaling to all images. Next, cross correlation was used to align the windowed images, and applied the measured shifts to all images. Finally, a moving median filter was used to produce a denoised time series in order to better identify candidate skyrmion locations.

Next, the spatial and temporal behavior of the skyrmion signals were analyzed. First, a Hough transform consisting of a 2D Gaussian shape (normalized by subtracting an error function profile to give the kernel a mean value of zero) was applied to each of the images, with a large range of radii. From the local maxima of the Hough transform over space global maxima as well as over time, we selected candidate skymion signals. We then fit a 2D Gaussian distribution to each of these candidates, over all time points. We used the scaling prefactor of the 2D Gaussian to identify skyrmions “turned ON” and “turned OFF”, i.e., which had prefactors that initially started near 0, then rose above +0.5 (or fell below −0.5 in the other domain), then fell back to near 0. From this subset of the skyrmions, we computed the diameter, the time between H ON and skyrmion creation, and the time between H OFF and skyrmion annihilation.

### Monte-Carlo simulations

The Monte Carlo simulations were carried out based on a two-dimensional model^51^, where exchange interaction, magnetic anisotropy, and the DMI are considered. The Hamiltonian is written as:

1 $${{{{{\mathscr{H}}}}}}{{{{{\mathscr{=}}}}}}{{{{{\mathscr{-}}}}}}J\,\mathop{\sum}\limits_{ < i,j > }{{{{{{\bf{S}}}}}}}_{i}\cdot {{{{{{\bf{S}}}}}}}_{j}-\mathop{\sum}\limits_{ < i,j > }{{{{{{\boldsymbol{\beta }}}}}}}_{{ij}}\cdot ({{{{{{\bf{S}}}}}}}_{i}\times {{{{{{\bf{S}}}}}}}_{j})-{K}_{{{{{{\rm{z}}}}}}}\mathop{\sum}\limits_{i}{\left|{{{{{{\bf{S}}}}}}}_{i,{{{{{\rm{z}}}}}}}\right|}^{2}$$

where ${{{{{{\bf{S}}}}}}}_{i}$ and ${{{{{{\bf{S}}}}}}}_{j}$ are spins located on sites $i$ and $j$ in a two-dimensional square lattice system. The dimensionless parameters $J$, $\beta (=|{{{{{{\boldsymbol{\beta }}}}}}}_{{ij}}|)$, ${K}_{{{{{{\rm{z}}}}}}}$ correspond to exchange interaction, DMI, magnetic anisotropy, respectively. The directions of ${{{{{{\boldsymbol{\beta }}}}}}}_{{ij}}$ are determined by $\hat{{{{{{\rm{z}}}}}}}\times {{{{{{\bf{r}}}}}}}_{{ij}}$, where ${{{{{{\bf{r}}}}}}}_{{ij}}$ is the distance vector between sites $i$ and $j$. The $< i,j >$ index pairs under the summations of exchange interaction and DMI refer to nearest neighbor pairs. To focus on the local switching behaviors of the skyrmions shown in our experiments, dipolar interaction is simply approximated as a shape anisotropy form. Thus, ${K}_{{{{{{\rm{z}}}}}}}$ can be considered as an effective anisotropy defined by the competition between the crystalline anisotropy of the system and the shape anisotropy induced by the dipolar interaction. Domain configurations shown in Fig. 4 are simulated using 1000$\times$100 lattice sites with periodic boundary condition applied for all directions (the center 1000$\times$50 area is shown in the figure), with the values of $J=1$, $\beta =0.3$, and ${K}_{{{{{{\rm{z}}}}}}}=0.40$ (initial state), $0.30$ (H ON state), $0.38$ (H OFF state). Eleven anisotropy defect sites with various ${K}_{{{{{{\rm{z}}}}}}}$ are placed in the highlighted area in Fig. 4a, where ${K}_{{{{{{\rm{z}}}}}},{{{{{\rm{site}}}}}}}$ are weaken by $\triangle {K}_{{{{{{\rm{z}}}}}},{{{{{\rm{site}}}}}}}=-0.05$ to $-0.25$ with a step of $0.02$. The diameter of these circle-like defects is 10 lattice sites. System temperature is applied by allowing spin fluctuations according to Boltzmann statistics. For each H ON or H OFF states, 100,000 iterations are performed to make the total energy stabilized, and the averaged spin configurations in the last 5000 iterations are shown in each cycle in Fig. 4.

## Supplementary information

Supplementary information

## Data Availability

The data that support the findings of this study are available from the corresponding authors upon reasonable request.
