# Supplementary material for: Reversible writing/deleting of magnetic skyrmions through hydrogen adsorption/desorption
Source: Nat Commun. 2022 Mar 15;13:1350. doi: 10.1038/s41467-022-28968-4 (PMC8924161; doi:10.1038/s41467-022-28968-4)
Supplement: Supplementary file 1 — Supplementary information [file 41467_2022_28968_MOESM1_ESM.pdf]

Supplementary Information

Reversible writing/deleting of magnetic skyrmions through hydrogen adsorption/desorption

Gong Chen[1,2*], Colin Ophus[3], Alberto Quintana[1], Heeyoung Kwon[4], Changyeon Won[5], Haifeng Ding[6], Yizheng Wu[7], Andreas K. Schmid[3], Kai Liu[1,2*]

[1] Physics Department, Georgetown University, Washington, DC 20057, USA
[2] Physics Department, University of California, Davis, California 95616, USA
[3] NCEM, Molecular Foundry, Lawrence Berkeley National Laboratory, Berkeley, California, 94720 USA
[4] Center for Spintronics, Korea Institute of Science and Technology, Seoul 02792, South Korea
[5] Department of Physics, Kyung Hee University, Seoul 02447, South Korea
[6] National Laboratory of Solid State Microstructures, Department of Physics and Collaborative Innovation Center of Advanced Microstructures, Nanjing University, 22 Hankou Road, Nanjing 210093, People's Republic of China
[7] Department of Physics, State Key Laboratory of Surface Physics and Advanced Materials Laboratory, Fudan University, Shanghai 200433, China

* Correspondence should be addressed to gchenncem@gmail.com (G.C.); Kai.Liu@georgetown.edu (K.L.)

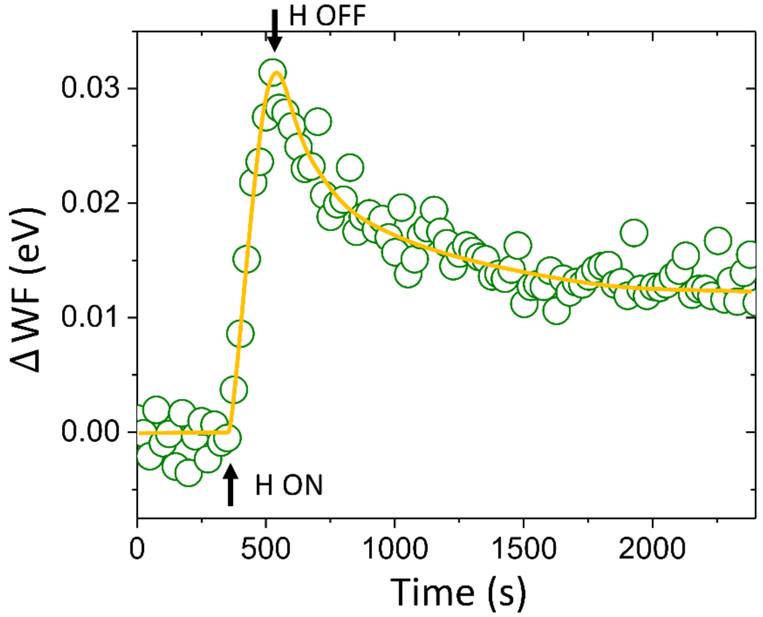

Figure S1. Evolution of the work function change ΔWF on the surface of 0.3ML Ni/3ML Co/4ML Pd/W(001) during the presence and absence of hydrogen at room temperature. Hydrogen "on" pressure is 5x10^{-9} torr. After switching hydrogen 'off' the work function does not fully revert to its initial value, i.e. ΔWF does not return to 0. This is related to hydrogen-coverage-dependent desorption kinetics discussed in Ref. [1], where desorption maxima β_1 (290-310 K, high hydrogen coverage saturating at 1ML) and β_2 (370-380 K, low hydrogen coverage saturating at 0.5ML) on the Ni(111) surface were revealed by the flash desorption [2,3]. Similar hydrogen desorption maxima were also found on the Co(0001) surface, where β_1 (325-370 K) and β_2 (400-420 K) were identified [4]. This irreversibility is also consistent with the small irreversibility of $|M_z|$ in Fig. 1j, where $|M_z|$ doesn't return to 1.

Supplementary Information

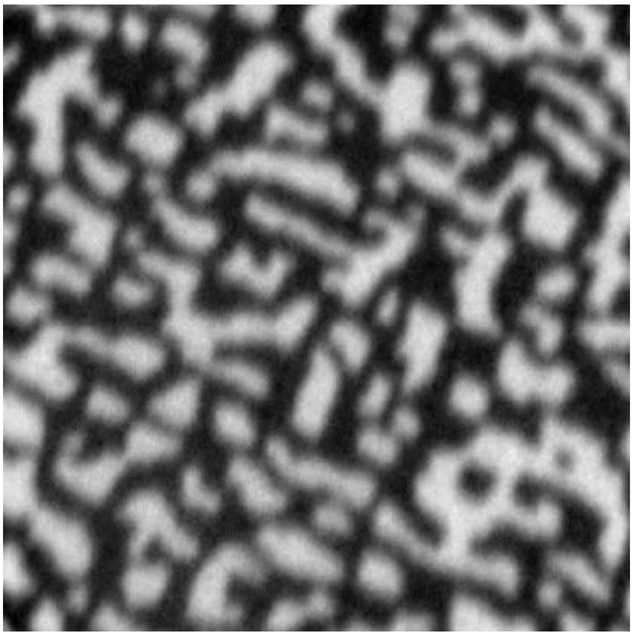

Figure S2. SPLEEM image with out-of-plane sensitivity of 24ML Ni/2ML Fe/1ML Ni/Cu(001), showing out-of-plane magnetized bubble-like domain pattern in the absence of magnetic field. The field of view is 7 μm. These bubble-like domains appear after the in-plane to out-of-plane spin reorientation transition at Ni thickness ~17 ML.

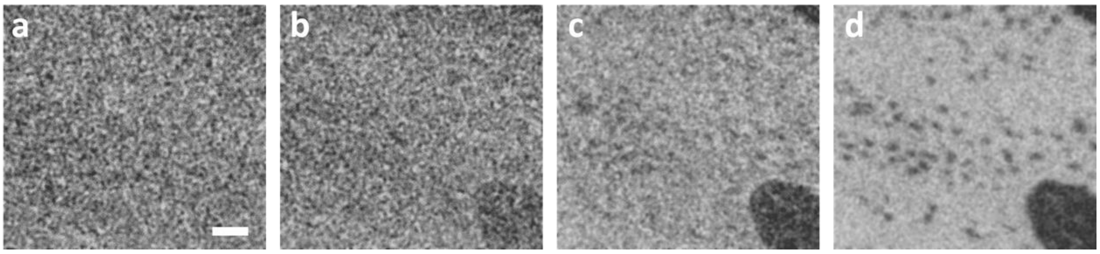

Figure S3. SPLEEM images with out-of-plane sensitivity as a function of Ni thickness d_{Ni} in Ni/3ML Co/5ML Pd/W(110), showing the evolution of out-of-plane magnetized domains during the SRT. (a) d_{Ni} = 0 ML, (b) d_{Ni} = 0.20 ML, (c) d_{Ni} = 0.27 ML, (d) d_{Ni} = 0.31 ML. Scale bar is 1μm. SPLEEM image in panel a contains a typical grey background without visible contrast, indicating that the film is in-plane magnetized. Out-of-plane magnetic contrast gradually develops in panels b-d, showing the details during the evolution.

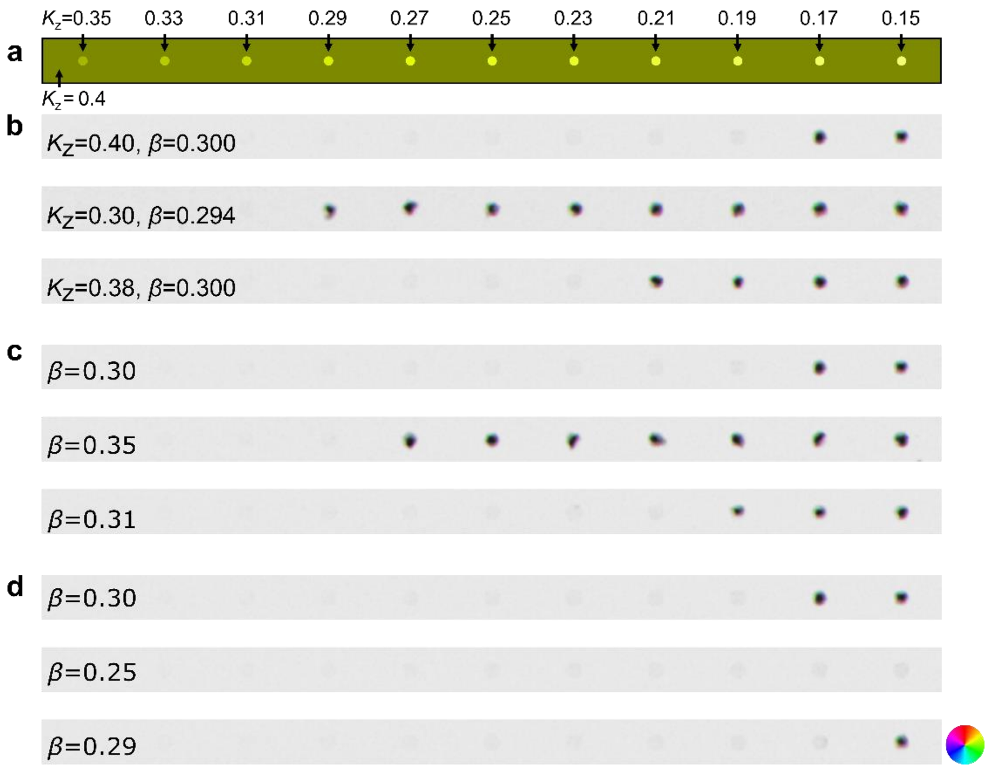

Figure. S4 Monte Carlo simulation of the effect of varying anisotropy K_z and DMI β, based on the same model used in Fig. 4. **a**, A sketch of the anisotropy landscape. **b**, Simulated domain evolution with additional small DMI change, showing that the small DMI change (decrease by 2%) is insufficient to affect the simulation results shown in Fig. 4 of the main text. **c**,**d**, Simulated domain evolution with DMI variation only, the anisotropy landscape is the same as Fig. 4a in the main text. Panel **c**, Skyrmion writing triggered by DMI increase. Panel **d**, skyrmion deleting triggered by DMI decrease.

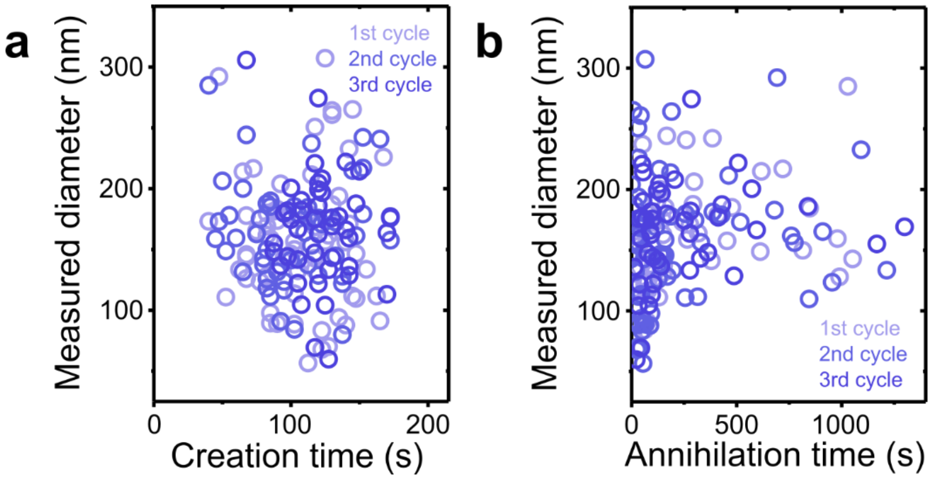

Figure S5. Relations between skyrmion diameter and the time required for skyrmion creation (panel a) and annihilation (panel b) over three cycles. The creation/annihilation time is counted from the instant when the hydrogen valve is turned ON/OFF until the moment each skyrmion appears/disappears. The creation/annihilation time is related to how fast chemisorption/desorption occurs at room temperature, which is evident in Fig. S1. The spread of the time values might be induced by the experimental variation of the anisotropy.

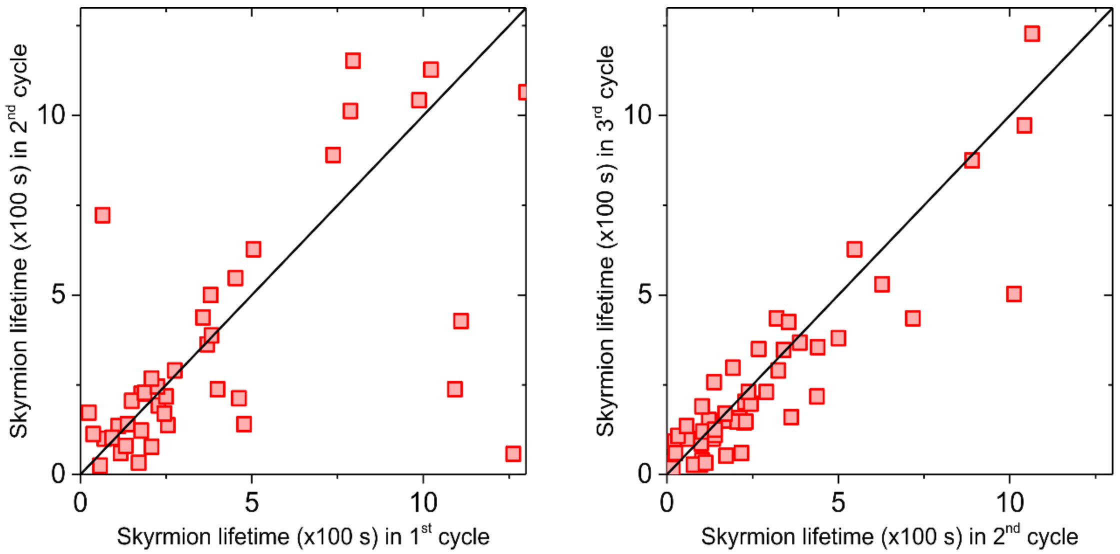

Figure S6. Comparison of skyrmion lifetimes between 1st/2nd (left panel) and 2nd/3rd (right panel) cycles. Black lines indicate equal skyrmion lifetime in the two successive cycles. Areas below/above the black line indicate longer/shorter skyrmion lifetime compared to the preceding cycle.

Supplementary Information

[1] G. Chen, M. Robertson, M. Hoffmann, C. Ophus, A. L. F. Cauduro, R. Lo Conte, H. F. Ding, R. Wiesendanger, S. Blügel, A. K. Schmid, and K. Liu, *Observation of hydrogen-induced Dzyaloshinskii-Moriya interaction and reversible switching of magnetic chirality*. Phys. Rev. X **11**, 021015 (2021).

[2] K. Christmann, O. Schober, G. Ertl, and M. Neumann, *Adsorption of hydrogen on nickel single crystal surfaces*. J. Chem. Phys. **60**, 4528 (1974).

[3] J. N. Russell, I. Chorkendorff, A. M. Lanzillotto, M. D. Alvey, and J. T. Yates, *Angular-Distributions of H-2 Thermal-Desorption - Coverage Dependence on Ni(111)*. J. Chem. Phys. **85**, 6186 (1986).

[4] Z. Huesges and K. Christmann, *Interaction of Hydrogen with a Cobalt(0001) Surface*. Z. Phys. Chem. **227**, 881 (2013).
